# Supplementary material for: Play vocalizations induce affective bias in reward learning and memory in orangutans
Source: Biol Lett. 2026 Oct 21;22(10):20260404. doi: 10.1098/rsbl.2026.0404 (PMC13682922; doi:10.1098/rsbl.2026.0404)
Supplement: Supplementary file 1 [file rsbl.2026.0404.zip › rsbl.2026.0404/rsbl20260404_si_001.docx]

Supporting information for

**Play vocalizations induce affective bias in reward learning and memory in orangutans**

**Authors**

Daan W. Laméris, Colin Allen, Heidi Lyn, Christopher F. Martin, Emma K. Nelson, Ximena J. Nelson, Alex Taylor, Erica A. Cartmill

Email: daan.lameris[at]kmda.org, ericac[at]iu.edu

This file contains:

Tables S1 to S5

Table S1: Orangutan participant demographics.

| **Participant** | **Sex** | **Date of birth** | **Species** | **Zoo** |
| --- | --- | --- | --- | --- |
| Azy | Male | 14-12-1977 | Bornean x Sumatran hybrid | Indianapolis Zoo |
| Bagus | Male | 17-06-2002 | Sumatran | ZOO Planckendael |
| Ombak | Male | 04-03-2017 | Sumatran | ZOO Planckendael |
| Vilmos | Male | 09-12-2010 | Sumatran | ZOO Planckendael |
| Karo | Female | 08-12-1973 | Sumatran | ZOO Planckendael |
| Moni | Female | 07-12-1977 | Sumatran | ZOO Planckendael |

Table S2: Example combinations of substrates used.

| **Substrate A** | **Substrate B** | **Substrate C** | **Location used** |
| --- | --- | --- | --- |
| Paper towel squares | Leaf litter | Fabric pieces | Indianapolis Zoo |
| Construction paper (green-black mix) | Yarn (orange) | Crepe paper (neutral) | Indianapolis Zoo |
| Wood wool | Carton shreds | Hemp | ZOO Planckendael |
| Moss | Yarn (yellow-blue mix) | Earth | ZOO Planckendael |
| Paper confetti | Cotton | Saw dust | ZOO Planckendael |

Table S3: Result of the quantity preference testing.

| **Participant** | **Score** | **P-value (one-tailed)** |
| --- | --- | --- |
| Bagus | 10/12 | 0.019 |
| Moni | 17/20 | 0.001 |
| Vilmos | 9/10 | 0.011 |
| Ombak | 11/12 | 0.003 |
| Karo | 10/12 | 0.019 |

Table S4: Individual-level preference scores and corresponding two-tailed binomial test p-values for the Reward Learning Assay. Choice biases are calculated as (proportion x 100) - 50.

| **Participant** | **Sum chosen target substrate** | **Proportion chosen target substrate** | **Choice bias** | **Binomial test p-value** |
| --- | --- | --- | --- | --- |
| Azy | 14 | 0.467 | -3.333 | 0.855 |
| Bagus | 25 | 0.833 | 33.333 | 0.0003 |
| Karo | 21 | 0.700 | 20.000 | 0.043 |
| Moni | 21 | 0.700 | 20.000 | 0.043 |
| Ombak | 17 | 0.567 | 6.667 | 0.585 |
| Vilmos | 22 | 0.733 | 23.333 | 0.016 |

Table S5: Individual-level preference scores and corresponding two-tailed binomial test p-values for the Affective Bias Test. Choice biases are calculated as (proportion x 100) - 50.

| **Participant** | **Sum chosen target substrate** | **Proportion chosen target substrate** | **Choice bias** | **Binomial test p-value** |
| --- | --- | --- | --- | --- |
| Azy | 16 | 0.533 | 3.333 | 0.855 |
| Bagus | 17 | 0.567 | 6.667 | 0.585 |
| Karo | 21 | 0.700 | 20.000 | 0.043 |
| Moni | 21 | 0.700 | 20.000 | 0.043 |
| Ombak | 23 | 0.767 | 26.667 | 0.005 |
| Vilmos | 23 | 0.767 | 26.667 | 0.005 |
